# What the elite athlete does not want to know

**DOI:** 10.1007/s12471-018-1082-8

**Published:** 2018-02-02

**Authors:** P. Sengkerij, M. E. W. Hemels

**Affiliations:** 1 Vitesse B.V., Arnhem, The Netherlands; 2 Department of Cardiology, Rijnstate Hospital, Arnhem, The Netherlands; 3 Radboud UMC, Nijmegen, The Netherlands

Sports is broadly integrated in our society. In early childhood specific sports can become a fascination, especially when the young person appears to be talented. At a certain stage only a select group gets the chance to become elite athletes and turn their hobby into a career. By that time, in most professional sports, several different interests and responsibilities begin to play a role. In that situation, it’s only logical that we perform some form of cardiovascular screening to evaluate the risk of sudden cardiac arrest in the athlete; it has a tremendous impact when it occurs. At the same time, however, important questions come up: Can we reliably predict the future for someone who has been identified with a cardiac disorder and, especially in the case of asymptomatic disorders, should this person be disqualified from competition, weighing the possible risks on life-threatening cardiovascular events on the one hand and the unique career perspectives on the other? Although sudden cardiac arrest is the leading cause of death among young athletes, we know little about the exact occurrence of sudden cardiac arrest due to structural heart disease in professional sports participants. Results of a large retrospective study performed in Canada (the Rescu Epistry cardiac arrest study, 3,825 persons included, 12–45 years of age) were recently published, reporting an incidence of 0.76 cases of sudden cardiac arrest during competitive sports per 100,000 athlete-years, with 43.8% of the athletes surviving until they were discharged from the hospital [1]. Based on the fact that in the 16 cases of sudden cardiac arrest that occurred during competitive sports only 2 cases of hypertrophic cardiomyopathy were identified, the authors concluded that sudden cardiac death during participation in competitive sports is rare and more than 80% of cases would not have been identified with systematic preparticipation screening. In this issue of the Netherlands Heart Journal Jorstad and Mosterd considerately debate the pros and cons, respectively, of preparticipation screening in young athletes [2, 3].

Recently, the European Heart Rhythm Association published a position paper on preparticipation cardiovascular evaluation for athletic participants to prevent sudden death [4]. The purpose of the position paper was to review the scientific evidence regarding the appropriate diagnostic methods to identify the cardiac conditions at risk in the athletic population. The paper further discussed the role of the different tests in the context of preparticipation cardiovascular evaluation. Furthermore, the consensus opinions relative to the most efficient preparticipation screening to improve safe sports participation according to current scientific evidence are described [4].

Currently, there are still important knowledge gaps in the optimal preparticipation screening programmes for elite sports participants and therefore it remains extremely difficult for the physician to interfere and eventually disqualify an athlete from competition. This decision has a huge, life-changing, impact on the individual athlete, their families and surroundings. Disqualifying an athlete from competition does have legal, financial, and mental consequences [5]. Therefore, as physicians we have to ask ourselves: Is the outcome of this decision in line with the Hippocratic oath, ‘Primum non nocere’ (first, do no further harm)? Are we in fact affecting the well-being of the individual athlete?

Although the incidence of sudden cardiac arrest is rare, we must continue with joint research to better predict the risks in athletes and minimise the occurrence of these tragic events in the future.
